# Supplementary material for: Predicting human protein function with multi-task deep neural networks
Source: PLoS One. 2018 Jun 11;13(6):e0198216. doi: 10.1371/journal.pone.0198216 (PMC5995439; doi:10.1371/journal.pone.0198216)
Supplement: S1 Table — This table reports all branch root terms, the number of GO terms in each branch, the names of branch root terms and their domains. (DOCX) [file pone.0198216.s001.docx]

**S1 Table. Summary of all branches.** This table reports all branch root terms, the number of GO terms in each branch, the names of branch root terms and their domains.

| Branch root term | #GO terms | Name | Domain |
| --- | --- | --- | --- |
| GO:0002376 | 18 | immune system process | BP |
| GO:0065007 | 218 | biological regulation | BP |
| GO:0009987 | 183 | cellular process | BP |
| GO:0008152 | 115 | metabolic process | BP |
| GO:0044699 | 189 | single-organism process | BP |
| GO:0000003 | 2 | reproduction | BP |
| GO:0032501 | 18 | multicellular organismal process | BP |
| GO:0051704 | 8 | multi-organism process | BP |
| GO:0040011 | 5 | locomotion | BP |
| GO:0022414 | 10 | reproductive process | BP |
| GO:0023052 | 3 | signaling | BP |
| GO:0071840 | 40 | cellular component organization or biogenesis | BP |
| GO:0032502 | 78 | developmental process | BP |
| GO:0040007 | 2 | growth | BP |
| GO:0050896 | 59 | response to stimulus | BP |
| GO:0051179 | 48 | localization | BP |
| GO:0022610 | 9 | biological adhesion | BP |
| GO:0007610 | 2 | behavior | BP |
| GO:0005215 | 19 | transporter activity | MF |
| GO:0005198 | 2 | structural molecule activity | MF |
| GO:0030234 | 8 | enzyme regulator activity | MF |
| GO:0001071 | 6 | nucleic acid binding transcription factor activity | MF |
| GO:0098772 | 9 | molecular function regulator | MF |
| GO:0005488 | 66 | binding | MF |
| GO:0000988 | 5 | protein binding transcription factor activity | MF |
| GO:0060089 | 7 | molecular transducer activity | MF |
| GO:0003824 | 44 | catalytic activity | MF |
| GO:0031974 | 3 | membrane-enclosed lumen | CC |
| GO:0032991 | 17 | macromolecular complex | CC |
| GO:0044456 | 2 | synapse part | CC |
| GO:0044425 | 18 | membrane part | CC |
| GO:0044421 | 3 | extracellular region part | CC |
| GO:0044422 | 29 | organelle part | CC |
| GO:0043226 | 28 | organelle | CC |
| GO:0016020 | 19 | membrane | CC |
| GO:0030054 | 6 | cell junction | CC |
| GO:0031012 | 2 | extracellular matrix | CC |
| GO:0044464 | 73 | cell part | CC |
| GO:0005576 | 2 | extracellular region | CC |
