# Supplementary material for: Predicting human protein function with multi-task deep neural networks
Source: PLoS One. 2018 Jun 11;13(6):e0198216. doi: 10.1371/journal.pone.0198216 (PMC5995439; doi:10.1371/journal.pone.0198216)
Supplement: S2 Table — *Different layers may have different number of hidden units. (DOCX) [file pone.0198216.s002.docx]

**S2 Table. Hyperparameters and their value space for MTDNN. *Different layers may have different number of hidden units.**

| **Hyperparameters** | **Value space** |
| --- | --- |
| The depth of shared hidden layers | [1, 2, 3] |
| The depth of specific hidden layers | [1, 2, 3] |
| The number of units in the shared hidden layers* | [300, 500, 600, 800] |
| The number of units in the specific hidden layers* | [60, 150, 300, 500] |
| Batch size | [50, 100, 150, 250] |
| The learning rate for the first stage | Log-uniformly selected from [0.01, 1] |
| The learning rate for the second stage | Log-uniformly selected from [0.01, 1] |
| Dropout rate | Uniformly selected from [0.25, 0.75] |
| Optimizer | ‘nesterov_momentum’ |
| Momentum (if applicable) | 0.9 |
| L1 regularization | [0, 0.0001, 0.00025, 0.0005, 0.00075, 0.0015, 0.005] |
| L2 regularization | [0, 0.0001, 0.00025, 0.0005, 0.00075, 0.0015, 0.005] |
