# Supplementary material for: Predicting human protein function with multi-task deep neural networks
Source: PLoS One. 2018 Jun 11;13(6):e0198216. doi: 10.1371/journal.pone.0198216 (PMC5995439; doi:10.1371/journal.pone.0198216)
Supplement: S4 Table — (DOCX) [file pone.0198216.s004.docx]

**S4 Table. The Spearman’s rank correlation coefficients between the branch size and performance improvement for three GO domains**

| **GO domain** | **Spearman’s rank correlation** |
| --- | --- |
| **BP** | **0.782** |
| **MF** | **0.400** |
| **CC** | **0.594** |
